# Introduction to the Special Issue on “State-of-the-Art Sensor Technology in Japan 2012”

**DOI:** 10.3390/s140611045

**Published:** 2014-06-23

**Authors:** Kouji Harada, Yoshiteru Ishida

**Affiliations:** Department of Computer Science and Engineering, Toyohashi University of Technology, 1-1 Hibarigaoka, Tempaku-cho, Toyohashi, Aichi 441-8580, Japan; E-Mail: harada@cs.tut.ac.jp

Since the previous special issue: State-of-the-Art Sensor Technology in Japan in 2008, which collected papers on sensing technology for monitoring of humans and the environment, we have experienced the Great East Japan Earthquake, Tsunami on 11 March 2011. This special issue, while aiming in the same direction, focuses on technologies for: (1) accuracy and sensitivity, (2) wireless functions, (3) real-time response, (4) portability (miniaturization), and (5) privacy preservation to promote sensor and sensing technologies for disaster prevention and resilient systems.

## Sensing Technologies: Yet another Perspective for Japan

In the previous special issue: State-of-the-Art Sensor Technology in Japan in 2008 [1], we focused on the perspectives that Japan faces: both human issues and environmental issues. Human issues originated from Japan's demographics, which are rapidly changing as the population ages. Environmental issues originated from Japan's international leadership focused on the global warming problem. However, after the Great East Japan Earthquake, Tsunami on 11 March 2011, and the Fukushima Daiichi nuclear plant accident, we realized the need to cover human-environment symbiotic issues.

One human-environment symbiotic issue is how to construct systems and societies that are resilient against natural and man-made disasters, while keeping constant efforts to maintain the natural environment. Resilient technologies including resilient sensing against disasters would be the responsibility of engineers and scientists. Sensors can gather data to detect disasters; recognize situations damaged by the disasters; and support decision-making for prompt responses to lessen the damage. Sensing technologies in Japan play a role in increasing resilience of sensors. The resilient sensors in turn help create and enhance resilient systems and community. We believe we can build a global sensor network with sensor systems installed at the gateway of the network for making the critical system resilient against disasters. For resilient sensing, sensors are required to detect dynamic and rapid changes in addition to static ones, microscopic changes as well as macroscopic ones, and private events and public ones. This special issue recognizes advancements of technologies for: (1) accuracy and sensitivity, (2) wireless functions, (3) real-time response (4) portability (miniaturization), and (5) privacy preservation depending on objects and environments for sensing. The present issue introduces state-of-the-art sensor technologies focusing on these five topics in Japan and finally proposes a ‘resilient’ technology.

## State-of-the-Art

Regarding the topics of accuracy and sensitivity, Ai [2] deals with a flight control mechanism of the silk moth, which has unique machinery for sensing dynamic changes in the natural environment. The paper taught us that the silk moth has developed a highly sensitive biosensor to detect its own wingbeats which are used as feedback information to accurately control its action during flight. Goto *et al.* [3], also dealing with a biosensor, investigated the potential of a peptide nucleic acid (PNA) probe instead of a commonly used DNA one for sensitively detecting single stranded DNA with its complementary sequence. They noted the lack of negative charges in the PNA backbone enhances the detection sensitivity of the PNA prove, although they impose a target for detection on the condition of its length is relatively short. Etoh *et al.* [4] review the history of technical progress in the field of *in situ* storage image sensors. They anticipated the frame rate of their proposing hexagonal CCD-type multi-collection gate (MCG) backside illumination (BSI) sensor will exceed one gigaframe per second in the near feature. When the technology is established, never-seen-before scenes would be unveiled for us. Zhou *et al.* [5] focuses on secure sensing technologies. Nowadays the number of digital equipment mounting fingerprint authentication methods instead of password is increasing, and the accuracy enhancement of fingerprint identification is strongly desired. To enhance the performance of standard fingerprint authentication algorithms based on SIFT descriptors, they proposed SIFT-based minutia descriptors (SMD) and succeeded in fingerprint identification with high accuracy. Shi *et al.* [6] deal with sensor technologies for local identification. To accurately identify the localization of a moving object, they propose a framework of SLAM with Bundle Adjustment utilizing GPS data. In the experimental test on a campus grounds using a vehicle equipped with the proposed method**,** they succeeded in identifying the location of the moving vehicle with an accuracy of several centimeters.

As for the wireless sensing, to synchronize multiple cameras without wiring them, Hou *et al.* [7] proposed a Manchester encoded illumination signal, whereby they open a new door for unwired vision sensor networks. Kan *et al.* [8] applied temperature-sensitive fluorescent dyes for a temperature sensor, enabling wireless temperature measurement in micro-regions. Fukuta *et al.* [9] developed a Laplacian electrode module to increase the sensitivity to electromyogram signal beneath the measurement site than conventional electrode modules. The newly developed electrode module has not only enhanced sensitivity but also wireless capability and portability thus extending applicability.

Regarding the field of real time sensing, Watanabe *et al.* [10] used 80-MHz repetition-rate femtosecond laser pulses and succeeded in analyzing terahertz time-domain polarization in real time. This technology will allow us to investigate low-energy dynamical phenomena in various materials in real time. Fujioka *et al.* [11] used an FF-2A electronic noise to realize a real time detection of the change of volatile patterns produced by *Aeromonas hydrophila*. Thus far, conventional detection methods for *Aeromonas hydrophila*, which induces a food contaminant, failed to detect the bacteria before the indication of the disease. This simple and real-time detection method is expected to help preventing food poisoning.

As for the topic of portable sensing, Tahara *et al.* [12] tackled taste sensors. Conventional taste sensing instruments were very heavy and thus not applicable for field use. In order to use it onsite, they developed a portable taste sensor device with a lipid/polimer membrane with a size comparable with a USB memory stick. Shimizu *et al.* [13] introduced a portable chemical decomposition system to improve indoor air quality (IAQ). They developed a portable microplasma reactor system to decompose formaldehyde indoors. To achieve its portability for the purpose of the use in a room environment, each microplasma electrode was devised to be on the order of micrometers in size.

As for the sensing private events, Tao *et al.* [14], aiming at privacy-preserved sensing in the home environment, developed an infrared ceiling sensor network system. The system encodes the existence/non-existence of a person as a binary value, so it can detect a person preserving his/her privacy. This system is expected to watch abnormalities of elders who live alone, while ensuring their privacy.

## Next Generation Sensors

We noted the bilateral character of sensor technology in the context of resilient technology. That is, sensor technology can enhance the resilience of the target system where sensors are installed; and sensors themselves must be resilient. To promote this direction, one possible avenue is *sensor systems* [1,15]. *Sensor systems* integrate many similar sensors like eyes, but also accommodate many distinct sensors like noses. Sensor systems can enhance the resilience of systems not only by locating and disconnecting faulty sensors but also by creating virtual sensors. They are not merely a collection of sensors; but are higher level sensing creations involving *in situ* information processing, just as multi-cellular organisms evolutionarily developed higher level sensing from simple reflective sensing.
